# Effect of Empagliflozin on Serum Levels of Thyroid Hormones Among Prediabetic and Diabetic Patients

**DOI:** 10.1155/ije/9920286

**Published:** 2025-04-17

**Authors:** Mojgan Sanjari, Narges Sadeghi, Ladan Amirkhosravi, Mohammad Hadavizadeh, Ahmad Naghibzadeh-Tahami, Zohreh Safi

**Affiliations:** ^1^Endocrinology and Metabolism Research Center, Kerman University of Medical Sciences, Kerman, Iran; ^2^Clinical Research Development Unit (CRDU), Sayad Shirazi Hospital, Golestan University of Medical Sciences, Gorgan, Iran; ^3^Gastroenterology and Hepatology Research Center, Institute of Basic and Clinical Physiology Sciences, Kerman University of Medical Sciences, Kerman, Iran; ^4^Social Determinants of Health Research Center, Institute for Futures Studies in Health, Kerman University of Medical Sciences, Kerman, Iran; ^5^Health Foresight and Innovation Research Center, Institute for Futures Studies in Health, Kerman University of Medical Sciences, Kerman, Iran; ^6^Physiology Research Center, Institute of Neuropharmacology, Kerman University of Medical Sciences, Kerman, Iran

**Keywords:** diabetes mellitus, empagliflozin, T3 thyroid hormone, T4 thyroid hormone, thyroid function tests

## Abstract

**Objectives:** Thyroid dysfunction (TD) and diabetes mellitus (DM) are the most prevalent chronic endocrine disorders that often coexist. Thyroid hormone affects glucose homeostasis through different mechanisms. SGLT2 inhibitors are a drug class used to treat diabetes. However, the effect of this drug class on thyroid tests has not been investigated among diabetic patients. Therefore, the current study aims to assess the effect of empagliflozin on thyroid tests.

**Materials and Methods:** This quasi-experimental study was conducted on 44 prediabetic and type 2 diabetic patients aged 18–65, directed to the clinic affiliated to Kerman Medical Sciences University in 2022–2023. Diabetic patients with HbA1c level of 0.5%–1% higher than the therapeutic target, who did not take blood sugar control drugs, were included in the study. All the patients received 10 mg of empagliflozin once daily for 3 months. Before and 3 months after taking empagliflozin, changes in TSH, total T4, and total T3 serum levels were measured.

**Results:** The mean patients' age was 54.77 years old. The mean FBS and HbA1c levels decreased 3 months after taking empagliflozin (*p* < 0.05). After taking empagliflozin, T3 level as well as T3/T4 ratio increased (*p*=0.001). However, no significant change was observed in T4 and TSH levels (*p* > 0.05). Also, level of T3 significantly changed with changes in weight and triglyceride level after taking empagliflozin.

**Conclusion:** The results showed taking empagliflozin could increase T3 level as well as T3/T4 ratio. However, it had no effect on total T4 and TSH levels.

**Trial Registration:** Iranian Registry of Clinical Trials (IRCT): IRCT20090317001774N10

## 1. Introduction

The prevalence of metabolic diseases has been increasing at a significant rate worldwide [1]. Thyroid dysfunction (TD) and diabetes mellitus (DM) are the most prevalent metabolic diseases [2]. As reported by International Diabetes Federation (IDF), more than 425 million people (9.1%) were diagnosed with diabetes worldwide in 2017 [3]. This disease is considered a global pandemic, as estimated to increase to more than 629 million people by 2045 [3]. Like diabetes, TD is also prevalent, so that its prevalence among the adults is 6.6% in Europe and the United States [4, 5].

Hypothyroidism and hyperthyroidism are more prevalent among type 2 DM patients (T2DM) than their nondiabetic peers [6, 7]. Centeno Maxzud et al. reported the prevalence of TD among T2DM patients to be 48.2% [8]. T2DM damages the conversion of thyroxine (T4) to triiodothyronine (T3) in peripheral tissues and lessens thyroid-stimulating hormone (TSH) levels. Poor management of T2DM could lead to hyperinsulinemia and insulin resistance, which increases nodule formation and goiter size and causes proliferation of thyroid tissue [5, 9]. Insulin-like growth factor-1 (IGF-1) is an important cell cycle progression factor and hypertrophic for all cell types. TSH, along with IGF-1 or insulin, stimulates proliferation and cell cycle progression in numerous thyroid cell culture systems [10]. Furthermore, circulating thyroid hormones impact different cells and organs and have a significant effect on protein, glucose, and lipid metabolism and could deteriorate glycemic control in T2DM [7]. Thyrotoxicosis and hyperthyroidism could worsen subclinical DM, provoke hyperglycemia among T2DM patients and increase the diabetic complications risk [6].

Antidiabetic drugs may affect thyroid function among T2DM patients. Metformin is one of the most commonly used oral drugs for T2DM treatment [11]. Metformin reduces TSH level and does not change free T3 and T4 levels in diabetic patients with primary hypothyroidism [12]. In prediabetic individuals, metformin diminishes the TSH serum level among those with TSH level greater than 2.5, lessens the small thyroid nodules size and precludes thyroid enlargement [13]. Thiazolidinediones (TZDs) are an important drug class utilized to treat diabetes that increases insulin sensitivity and acts by raising the Peroxisome proliferator-activated receptors (PPARs) activity [14]. TZDs are PPARγ agonists, which are frequently seen in the patients thyroid tissue with Graves' and disease thyroiditis [14] as well as in the eye patients tissue with thyroid eye disease (TED) [15]. Exacerbation of proptosis and TED following treatment with TZD has been reported among some T2DM patients [15]. GLP-1 agonists decrease TSH and increase the risk of medullary carcinoma in laboratory rats [16]. Taking the first-generation sulfonylurea increases the prevalence of hypothyroidism [17]. Limited information is available regarding the effect of acarbose and sodium-glucose cotransporter-2 (SGLT2) inhibitor drugs on thyroid function. Acarbose has been associated with increased thyroid hormones in diabetic rats. A limited number of studies have been conducted on SGLT2 inhibitors [18].

SGLT2 inhibitors are a new anti-diabetic drug class that reduces blood sugar through glucosuria and reducing weight, insulin resistance and hepatic steatosis, and increases energy consumption by oxygen consumption and CO_2_ production [19]. SGL2 inhibitors have a significant impact on metabolic indices, including body fat percentage, body mass index (BMI) and, waist circumference and have renal protective effects and cardiovascular [20]. Empagliflozin significantly affects arterial stiffness, albuminuria and plasma urate and increases low-density lipoprotein (LDL) and high-density lipoprotein (HDL) levels [21, 22]. Okada et al. in a multicenter and retrospective observational study reported that in the group receiving SGL2 inhibitors FT3 and FT3/FT4 ratio among T2DM patients were higher than the levels in the group not treated with SGL2 inhibitors. Also, FT4 level was lower in this group. However, the level of TSH was not difference between two groups [23].

Various studies have been conducted on empagliflozin. Due to the limited investigation of the effect of this drug class on thyroid tests, the current research aims to evaluate the empagliflozin effect on thyroid function tests among diabetic and prediabetic patients.

## 2. Materials and Methods

### 2.1. Subjects

This quasi-experimental study was conducted on prediabetic patients or patients with definite diagnosis of DM, who aged 18–65 years old and referred to one of the clinics affiliated to Kerman University of Medical Sciences in 2021-2022. Also, they were a member of Kerman coronary artery diseases risk factors study (KERCADRS). After obtaining the required approval from Ethics Committee of Kerman University of Medical Sciences (IR.KMU.AH.REC.1401.224), the patients were selected considering the exclusion and inclusion criteria. The procedures of study were described to the patients, and a written informed consent form was taken from them to enter the study.

The inclusion criteria were being 18–65 years old, suffering from prediabetes or diabetes with HbA1c level of maximum 0.5%–1% higher than the appropriate treatment goal of the patient and not taking blood sugar control drugs. It should be noted that prediabetes and diabetes were defined based on the criteria provided by the American Diabetes Association. Meeting one of the following criteria was considered as having prediabetes: Laboratory glucose level of 100–125 mg/dL after 8 h of fasting and two-hour laboratory glucose level of 140–199 mg/dL (2 h after consuming 75 g of oral glucose). Meeting one or two of the following criteria was considered as having diabetes: Glucose level equal to or higher than 126 mg/dL and level of glucose equal to or greater than 200 mg/dL.

The exclusion criteria were a history of hypothyroidism or hyperthyroidism, abnormal thyroid tests, taking levothyroxine or methimazole, suffering from type 1 DM (T1DM), taking oral or injectable drugs controlling glucose levels (insulin, biguanides, sulfonylureas, TZDs, GLP-1 receptor agonists, SGLT2 inhibitors, bromocriptine or cholestyramine), taking drugs that affect the metabolism of thyroid hormones such as amiodarone, lithium, androgens, estrogen, etc., a history of diabetic ketoacidosis, glomerular filtration rate (GFR) less than 30, suffering from underlying diseases that predispose one to acidosis, having liver failure and taking biotin.

**Figure 1:**
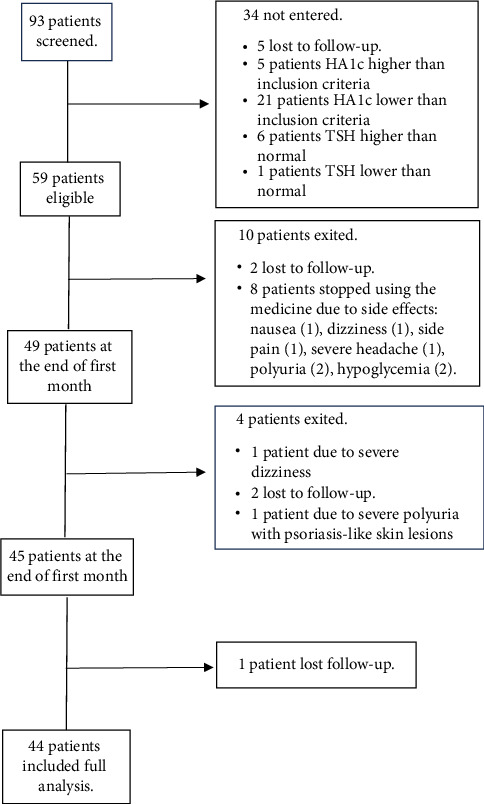
The flowchart shows the recruitment of the study subjects.

In this study, 93 patients were included in the study in the order of admission. Among all, 34 patients did not meet the inclusion criteria after performing the initial tests and were excluded, five of whom did not refer after the initial screening despite making multiple phone calls. Also, 5 and 21 individuals were excluded due to having HbA1c level higher and lower than the inclusion criterion, respectively, 6 individuals were excluded due to TSH higher than the normal range and one person was excluded due to TSH lower than the normal range. Also, 10 patients were excluded during the first month: Two patients were excluded due to nonreferral despite making multiple calls and eight patients were excluded because of stopping drug consumption due to its side effects (nausea (1 patient), flank pain (1 patient), polyuria (2 patients), sugar drop based on the patient's statements and glucometer (2 patients), dizziness (1 patient), severe headache (1 patient). Out of the remaining 49 patients, four patients were excluded from the study during the second month: one person due to severe dizziness, two patients due to lack of cooperation and nonreferral and one patient due to severe polyuria with psoriasis-like skin lesions and lower limb edema. Also, one patient did not refer after 3 months of receiving the medicine despite making several phone calls. Finally, 44 patients (22 female and 22 male) completed the follow-up period (Figure 1).

### 2.2. Anthropometric Measurements

After entering the study, patients' characteristics, including their demographic and personal characteristics, were recorded using the data extraction form. Patients' weight was measured using a digital scale (Omron) with the accuracy of 100 g with minimal clothing without shoes and their systolic and diastolic blood pressure was obtained after a 15 min rest while sitting in the clinic.

### 2.3. Interventions

The patients were asked to go to the clinic laboratory between 8 and 10 a.m. on the specified day after fasting for 12 h to take blood samples. Thus, 10 mL of blood was collected from the patients and stored in −80°C at Kerman Endocrine and Metabolism Research Center laboratory. Then, all the patients received 10 mg of empagliflozin (manufactured by Dr. Abidi's pharmaceutical company, Iran, under the brand name Gloripa) once daily for 3 months. The drug was prescribed monthly. At the end of each month, the patients were examined in terms of side effects and drug use continuation as well as height, weight and blood pressure. At the end of 3 months, the patients were recalled going to the clinic laboratory between 8 and 10 a.m. on the specified day after fasting for 12 h to take blood samples. The blood samples were stored at −80°C. At the end of 3 months, the blood samples were assayed in terms of serum levels of T4 (total), T3 (total), TSH, creatinine (Cr), total cholesterol, triglyceride (TG), LDL, HDL, FBS, and HbA1c in the same conditions as before receiving the drug.

### 2.4. Outcomes

In this study, the primary outcomes included changes in TSH serum levels, total T3 and total T4. The secondary outcomes included changes in weight, systolic and diastolic blood pressure levels, and serum levels of Cr, total cholesterol, TG, LDL, and HDL which will be reported in more detail in another paper [24].

### 2.5. Laboratory Analyses

Serum tests of thyroid function were examined utilizing the immunoassay system with an automated analyzer Siemens 2010 (ADVIA Centaur CP Immunoassay System). The plasma level of glucose was measured by glucose oxidase technique (using Pars Azmoun kit, Iran). Also, HbA1c was determined using standard laboratory kits (Pars Azmoun Co., Iran).

## 3. Statistical Analysis

The data were represented as mean ± standard deviation (SD). Changes in the mean parameters of the study were investigated by the paired samples *t*-test or its nonparametric version, i.e., Wilcoxon, considering the normal or non-normal distribution of the data. Also, general estimating equation (GEE) was used to investigate the relationship between thyroid hormones and other variables before and after taking empagliflozin. The significance level was set to 95% and *p* value less than 0.05. Analysis of statistics was accomplished using STATA version 17.

## 4. Results

The mean age of the patients (*n* = 44) who entered the study was 54.77 years old (Table 1), 22 of whom were female and 22 were male. As indicated in Table 1, the mean patients' weight was 77.65 ± 15.97 kg at the beginning of the study, which decreased by 3 kg (4.05%) after receiving empagliflozin (*p* < 0.001). Also, the mean FBS was 115.11 ± 28.7 mg/dL before the study, which significantly decreased by 8.5 mg/dL after receiving empagliflozin (*p*=0.04) and reached 106.68 ± 26.58 mg/dL. Moreover, the mean HbA1c level significantly decreased after receiving empagliflozin (*p*=0.031).

The mean TSH level was 2.47 ± 2.02 mlU/L at the beginning of the study, which decreased by 0.227 (9.16%) after receiving empagliflozin (2.25 ± 1.58 mlU/L), which was not statistically significant (*p*=0.38) (Table 2). The mean T3 level was 0.95 ± 0.18 ng/dL at the beginning of the study, which was significantly increased by 0.132 (13.8%) after receiving empagliflozin drug and reached 1.092 ± 0.23 ng/dL (*p* < 0.001). The mean T4 level was 7.88 ± 1.33 ng/dL at the beginning of the study, which increased by 0.225 (2.85%) after receiving empagliflozin drug and reached 8.11 ± 1.55 ng/dL. Although, this increase was not statistically significant (*p*=0.201). The mean T3/T4 ratio was 0.12 ± 0.02 at the beginning of the study, which increased by 0.13 (10.94%) after receiving empagliflozin and reached 0.13 ± 0.02, which was statistically significant (*p* < 0.001) (Table 2).

Table 3 displays the relationship between T3 and other variables after taking empagliflozin. T3 level changes with changes in weight and triglyceride, so that T3 level increased by 0.007 μgr/mL per kg of weight change (*p* < 0.001). T3 level changed by 0.001 µgr/mL per 1 mg/dL change in triglyceride level (*p*=0.006). Other variables had no significant effect on T3 level (*p* > 0.05).  Table 4 presents the relationship between HDL and thyroid tests. HDL level significantly changed with TSH change (*p*=0.039). HDL level increased by 0.64 mg/dL per 1 mlU/L change in TSH.

## 5. Discussion

DM and TD are among the most prevalent chronic endocrine disorders with variable prevalence among different populations, which are seen together [25, 26]. Numerous studies have demonstrated high TD prevalence among diabetic patients and vice versa. Thyroid hormones affect homeostasis of glucose by different mechanisms [17, 27]. Previous studies have reported the effect of other antidiabetic drugs on thyroid function [17, 28]. The current research assayed the effect of SGL2 inhibitors on thyroid function among prediabetic and diabetic patients. The results showed taking empagliflozin 10 mg for 3 months significantly increased T3 level. Also, TSH level decreased, but it was not statistically significant. Moreover, T3/T4 ratio significantly increased. After taking empagliflozin, the T3 level significantly increased with changes in weight and triglyceride.

Okada et al. conducted a retrospective study to investigate thyroid function among T2DM patients in 2019–2021. In line with our study, they reported that TSH level decreased in the group receiving SGLT2 inhibitor, which was not significant. Also, T3 level significantly increased and T3/T4 ratio was significantly greater. However, T4 level significantly decreased, which was in contrast to our observations [23]. Also, inconsistencies were observed in the results of the two studies. The present research was a prospective clinical trial. The patients who were examined in the study by Okada received other diabetic drug classes except SGLT2 inhibitor, which could affect the results. In Okada's study, patients in the group receiving SGLT2 inhibitor had significantly higher T3, lower T4, higher T3/T4 ratio and higher BMI from the beginning and were younger. There was no significant difference between the two groups in other characteristics, including blood pressure, lipid and Cr [23].

Previous studies have revealed SGLT2 inhibitors could indirectly affect the activity of iodothyronine deiodinase (DIO) and circulating T3 is mostly derived from T4 deiodination. Also, SGLT2 inhibitors improves DIO2 activity in the adipose tissue of rodents and humans [29, 30]. Therefore, it seems that SGLT2 inhibitors may increase the T3/T4 ratio in this way, which was observed in our research as well as Okada's study. Besides, SGLT2i enhances function of renal [31, 32] and the most of circulating T3 is derived from deiodination of T4 by DIO1 activity in the thyroid, kidney, and liver [33]. An animal study showed that treating diabetic rats with T3 reduces SGLT2 expression in the kidney and increases glucose excretion in urine. Consequently, levels of thyroid hormones may perhaps change the SGLT2i effect on the control of blood sugar in diabetes patients [34]. Moreover, it is conceivable that empagliflozin's effects on muscle mass could have contributed to the observed rise in T3 levels, given the documented link between thyroid hormones and skeletal muscle metabolism [35, 36]. Since skeletal muscle is a key location for T4-to-T3 conversion through deiodinase activity [37, 38], alterations in muscle mass or metabolism may have an effect on the levels of T3 in the blood.

Furthermore, it has been reported that SGLT2 inhibitor (canagliflozin) inhibits glucose uptake, glycolysis and AKT/mTOR signaling activation in patients with PTC and increases activation of adenosine monophosphate-activated protein kinase (AMPK) and apoptosis in thyroid cancer cells, thereby reducing growth of thyroid cancer cells in vivo and in vitro [39]. Moreover, the empagliflozin effect on thyroid tests could be investigated through AMPK pathway, which is one of the biological pathways for maintaining energy homeostasis and regulating metabolism [40]. Also, AMPK is a central target for the insulin sensitivity modulation and the feedback of thyroid hormones on energy expenditure and appetite [6]. This pathway is involved in drug treatments and combating insulin resistance, metabolic disorders and obesity [41, 42]. SGLT2 inhibitors act on AMPK similar to metformin, and the effects of empagliflozin on thyroid hormones may be similar to those of metformin [41, 42]. Metformin passes through the blood-brain barrier and the TSH inhibition central mechanism by metformin could justify changes in the thyroid axis [11, 40]. Metformin activates AMPK in the periphery, but suppresses AMPK activity in the hypothalamus and possibly counteracts hypothalamic T3 action on TSH secretion [11, 43]. Inhibition of AMPK activity in the hypothalamus strengthens the inhibitory feedback of thyroid hormones on TSH secretion [40]. These effects do not change TSH level if the feedback system works properly [44]. T3 level is associated with a significant thermogenic response, increases sympathetic activity and suggests another mechanism for weight loss [43, 45]. Other hypotheses raised to make changes in thyroid tests include changes in the thyroid hormone receptors affinity, bioavailability, TH binding, metabolism, activation of TSH receptor and TSH assay-interference [9, 12].

The strengths of this study included the prospective design and excluding patients who were taking other blood sugar control drugs. Reviewing the literature revealed not many studies have investigated the effect of empagliflozin on thyroid function and tests. Thus, this is among the pioneering studies in this field. In patients taking this drug, changes in the normal range of thyroid tests could be expected, which is not considered pathological and does not require intervention. Being aware of this issue makes both physicians and patients more confident to take this drug. Finally, the present study showed taking empagliflozin could increase T3 and T3/T4 ratio, but it had no effect on total T4 and TSH levels. Given the high co-existence of subclinical hypothyroidism and DM [2], some hypothyroidism patients have a low quality of life on levothyroxine treatment despite full treatment and normal TSH [46, 47]. Some authors recommended adding T3 to therapy in such cases [48]. Based on the above data, it may be reasonable that empagliflozin could be a part of treatment in patients who have DM and hypothyroidism. This needs further investigation.

## Figures and Tables

**Table 1:** Subjects' characteristics at baseline and 3 months after treatment.

Variable	Baseline	3 Months	*p* value
BW (kg)⁣^∗^	77.65 ± 15.97	74.5 ± 15.03	< 0.001
SBP (mmHg)	136.45 ± 15.37	132.68 ± 18.21	0.061
DBP (mmHg)	82.31 ± 10.29	83.7 ± 9.61	0.131
BMI (kg/m2)	29.66 ± 4.19	28.47 ± 2.94	0.65
Waist circumference(cm)	101.79 ± 9.11	99.35 ± 7.95	0.42
FBS (mmol/L)⁣^∗^	115.11 ± 28.7	106.68 ± 26.58	0.041
Hb A1c (%)⁣^∗^	6.53 ± 0.68	6.38 ± .75	0.031
BUN (mg/dL)	25.43 ± 6.12	26.15 ± 5.85	0.350
Cr (mg/dL)⁣^∗^	0.8 ± 0.15	0.88 ± 0.14	< 0.001
GFR (mL/min)⁣^∗^	108.21 ± 31.03	94.36 ± 3.86	< 0.001
TG (mg/dL)	157.04 ± 70.85	143.52 ± 79.43	0.207
Cholesterol (mg/dL)	126.41 ± 32.67	133.79 ± 28.04	0.220
HDL (mg/dL)⁣^∗^	48.31 ± 8.27	51.93 ± 6.44	0.013
LDL (mg/dL)	103.97 ± 26.88	113.99 ± 37.08	0.156

*Note:* Data are expressed as mean ± SD. Cr: creatinine, HbA1c: glycated hemoglobin; TG, triglyceride.

Abbreviations: BMI, body mass index; BUN, blood urea nitrogen; BW, body weight; DBP, diastolic blood pressure; GFR, glomerular filtration rate; HDL, high-density lipoprotein; LDL, low-density lipoprotein; SBP, systolic blood pressure.

⁣^∗^*p* < 0.05.

**Table 2:** Effect of 3 months empagliflozin use on thyroid hormones.

Variable	Baseline	3 Months	*p* value
TSH (mIU/mL)	2.47 ± 2.02	2.25 ± 1.58	0.386
T3 (pg/mL)⁣^∗^	0.95 ± 0.18	1.09 ± 0.23	< 0.001
T4 (ng/dL)	7.88 ± 1.33	8.11 ± 1.55	0.201
T3/T4 ratio⁣^∗^	0.12 ± 0.02	0.13 ± 0.025	< 0.001

*Note:* Data are expressed as mean ± SD. T3: triiodothyronine; T4: thyroxine.

Abbreviation: TSH, thyroid stimulating hormone.

⁣^∗^*p* < 0.05.

**Table 3:** Association of T3 with other variables after 3 months empagliflozin use.

Variable	β	*p* value
BW (kg)⁣^∗^	0.007	< 0.001
SBP (mmHg)	−0.017	0.997
DBP (mmHg)	−0.037	0.680
FBS (mmol/L)	0.00	0.679
HbA1c (%)	−0.039	0.37
BUN (mg/dL)	0.001	0.77
Cr (mg/dL)	0.078	0.679
TG (mg/dL)⁣^∗^	−0.001	0.006
Cholesterol (mg/dL)	−0.001	0.257
HDL (mg/dL)	−0.003	0.355
LDL (mg/dL)	−0.001	0.110

*Note:* T3 level changes with changes in weight and triglyceride. HbA1c: glycated hemoglobin; Cr: creatinine; TG, triglyceride.

Abbreviations: BUN, blood urea nitrogen; BW, body weight; DBP, diastolic blood pressure; FBS, fasting blood glucose; GFR, glomerular filtration rate; HDL, high-density lipoprotein; LDL, low-density lipoprotein; SBP, systolic blood pressure.

⁣^∗^*p* < 0.05.

**Table 4:** Association of HDL with thyroid hormones after 3 months empagliflozin use.

Variable	*β*	*p* value
T3	−0.003	0.355
TSH⁣^∗^	0.640	0.039
T4	−0.319	0.635

*Note:* The HDL level significantly changed with TSH change. T3: triiodothyronine; T4: thyroxine.

Abbreviation: TSH, thyroid stimulating hormone.

⁣^∗^*p* < 0.05.

## Data Availability

The data that support the findings of this study are available from the corresponding author upon reasonable request.
